# METTL3 regulates alternative splicing of MyD88 upon the lipopolysaccharide‐induced inflammatory response in human dental pulp cells

**DOI:** 10.1111/jcmm.13491

**Published:** 2018-03-04

**Authors:** Zhihui Feng, Qimeng Li, Runsha Meng, Baicheng Yi, Qiong Xu

**Affiliations:** ^1^ Guanghua School of Stomatology Guangdong Provincial Key Laboratory of Stomatology Sun Yat‐sen University Guangzhou China

**Keywords:** N6‐methyladenosine, METTL3, alternative splicing, MyD88, lipopolysaccharide, dental pulp inflammation

## Abstract

Dental pulp inflammation is a widespread public health problem caused by oral bacterial infections and can progress to pulp necrosis and periapical diseases. N6‐methyladenosine (m6A) is a prevalent epitranscriptomic modification in mRNA. Previous studies have demonstrated that m6A methylation plays important roles in cell differentiation, embryonic development and stress responses. However, whether m6A modification affects dental pulp inflammation remains unknown. To address this issue, we investigated the expression of m6A and N6‐adenosine methyltransferase (METTL3, METTL14) as well as demethylases (FTO, ALKBH5) and found that the levels of m6A and METTL3 were up‐regulated in human dental pulp cells (HDPCs) stimulated by lipopolysaccharide (LPS). Furthermore, we knocked down METTL3 and demonstrated that METTL3 depletion decreased the expression of inflammatory cytokines and the phosphorylation of IKKα/β, p65 and IκBα in the NF‐κB signalling pathway as well as p38, ERK and JNK in the MAPK signalling pathway in LPS‐induced HDPCs. The RNA sequencing analysis revealed that the vast number of genes affected by METTL3 depletion was associated with the inflammatory response. Previous research has shown that METTL3‐dependent N6‐adenosine methylation plays an important role in mRNA splicing. In this study, we found that METTL3 knockdown facilitated the expression of MyD88S, a splice variant of MyD88 that inhibits inflammatory cytokine production, suggesting that METTL3 might inhibit the LPS‐induced inflammatory response of HDPCs by regulating alternative splicing of MyD88. These data shed light on new findings in epitranscriptomic regulation of the inflammatory response and open new avenues for research into the molecular mechanisms of dental pulp inflammation.

## Introduction

N6‐methyladenosine (m6A), methylation at the N6 position of adenosine, has been identified as the most abundant epitranscriptomic modification in eukaryotic mRNA since its discovery in the 1970s 1, 2, 3. However, the distribution of m6A remains a mystery due to the lack of effective techniques and its function has been largely absent over the past few decades 4. Recent studies used an m6A RNA immunoprecipitation approach followed by high‐throughput sequencing and demonstrated that m6A is enriched around stop codons, in 3′ untranslated regions (3′UTRs) and within internal long exons 4, 5. All m6A sites are found within sequences conforming to the consensus sequence RRm6ACH ([G/A/U][G>A]m6AC[U>A>C]) 6. N6‐adenosine methylation is installed by an m6A methyltransferase complex including methyltransferase‐like 3 (METTL3), METTL14 and the regulatory subunit Wilms tumour 1‐associated protein (WTAP), with S‐adenosyl‐methionine (SAM) as the methyl donor 7. The removal of m6A is facilitated by fat mass and obesity‐associated protein (FTO) and alkB homologue 5 (ALKBH5) 8, 9. Thousands of mRNAs in human cells are subject to m6A modification, which affects almost every stage of mRNA metabolism, including pre‐mRNA splicing, mRNA transport from the nucleus to cytoplasm, translation, mRNA turnover, mRNA stability and subcellular localization 10.

Dental pulp inflammation is a typical inflammatory disease characterized by local accumulation of inflammatory mediators in dental pulp, and this inflammation can progress to pulp necrosis and periapical diseases 11, 12. Bacterial infection is usually represented as the most important aetiological factor in pulpal and periapical diseases 13. Lipopolysaccharide (LPS) from the cell walls of Gram‐negative bacteria can penetrate the pulp and trigger the inflammatory response 14. LPS is thought to promote cytokine/chemokine expression by binding to toll‐like receptors (TLR4 and/or TLR2), which engage either myeloid differentiation primary response gene 88 (MyD88)‐dependent or MyD88‐independent pathways 15, 16, 17. MyD88 recruits IL‐1R‐associated kinase‐4 (IRAK4), which subsequently recruits, activates and degrades IRAK1. IRAK1 then combines with TNF‐receptor‐associated factor‐6 (TRAF6) and activates the nuclear factor‐κB (NF‐κB) and mitogen‐activated protein kinase (MAPK) pathways 18, which play crucial roles in the induction of inflammatory mediators 19. TLR/MyD88‐mediated NF‐κB and MAPK signalling pathways are reported to be activated in LPS‐induced dental pulp inflammation 20. MyD88 is encoded by an mRNA with five exons (MyD88L) and produces different splice variants through alternative splicing 21. A shorter MyD88 mRNA (MyD88S), lacking the second exon, exerts a dominant‐negative effect on LPS‐induced, TLR4‐mediated signalling pathways and inhibits inflammatory cytokine production 22, 23.

Recent studies have reported that m6A methylation plays important roles in a variety of human disorders and diseases, such as obesity, cancer, type 2 diabetes mellitus, infertility and developmental arrest 24, 25, 26, 27. However, it is unknown whether m6A modification can affect the development of dental pulp inflammation. Whether m6A affects the LPS‐induced inflammatory response in HDPCs by regulating the alternative splicing of MyD88 also remains to be determined. Therefore, the aims of this study were to investigate the effect of m6A methylation on LPS‐induced inflammatory responses in HDPCs and to determine the underlying molecular mechanism.

## Materials and methods

### Cell culture

This study was approved by the Ethical Review Board of the Guanghua School of Stomatology of Sun Yat‐sen University. All participants in this study gave written informed consent. HDPCs were isolated and cultivated as previously described by Gronthos *et al*. 28. Briefly, pulp tissues from freshly extracted normal human impacted third molars (18–25 years of age) were gently separated from the crown and root and then digested in 3 mg/ml collagenase type I (Gibco, Grand Island, NY, USA) for 20 min at 37°C. Subsequently, the pulp tissues were placed in 25‐cm^2^ culture flasks containing Dulbecco's modified Eagle's medium (DMEM) supplemented with 100 U/ml penicillin, 100 U/ml streptomycin and 20% foetal bovine serum (FBS) (Gibco) and incubated at 37°C in an atmosphere of 95% O_2_ and 5% CO_2_. The media were changed every 3 days. When the cells reached 80% confluence, they were harvested using trypsin/EDTA (Gibco) and subcultured at a ratio of 1:3. Cells from passages 2 to 4 were used for further study.

### LPS stimulation and total m6A measurement

The cells were cultured in six‐well culture dishes until they reached approximately 80% confluence and were then treated with 1 μg/ml *Porphyromonas gingivalis* LPS (InvivoGen, San Diego, CA, USA) for 3, 6, 12 and 24 hrs. Cells without LPS stimulation were used as controls.

Total RNA was extracted, and total m6A content was determined using an m6A RNA methylation quantification kit (EpiGentek, Farmingdale, NY, USA) according to the manufacturer's instructions.

### METTL3 knockdown using siRNA transfection

A siRNA strategy was used to knock down METTL3 in HDPCs. Briefly, 1 × 10^5^ cells were added to fresh medium without antibiotics and seeded in six‐well plates 24 hrs before transfection. For transfection, all siRNAs (siMETTL3 or the negative control, i.e. siCTRL) (Invitrogen, Carlsbad, CA, USA) were resuspended at a concentration of 20 μM and then transfected into HDPCs using Lipofectamine^®^ RNAiMAX transfection reagent (Invitrogen) when the cells reached 70% confluence. A transfection rate of 70–85% of cells was used for further experiments.

### Quantitative real‐time PCR (qPCR) and reverse transcription PCR (RT‐PCR) monitoring of mRNA levels

Total RNA from HDPCs was extracted using TRIzol reagent (Invitrogen) according to the manufacturer's instructions. One microgram of RNA from each sample was reverse‐transcribed for cDNA synthesis using Superscript III Reverse Transcriptase (Invitrogen). Subsequently, the cDNA was used as a template for PCR. qPCR was conducted using a LightCycler 480 with SYBR green I master mix (Roche, Basel, Switzerland).

To determine the expression of MyD88 splice variants, semi‐quantitative RT‐PCR followed by agarose gel electrophoresis was performed. One microgram of RNA, prepared as described above, was first subjected to reverse transcription using Superscript III Reverse Transcriptase (Invitrogen). The reverse‐transcribed cDNA was then subjected to PCR using Taq DNA polymerase (Invitrogen). PCR products were electrophoresed using 2% agarose gels, and images were captured using the FluorChem^™^ Q system (Alpha Innotech, San Jose, CA, USA). All primer sequences are listed in Table 1.

**Table 1:** Primer sequences for qRT‐PCR and RT‐PCR

Gene	Forward primer(5′–3′)	Reverse primer(5′‐3′)	Notes
METTL3	GAGGAGTGCATGAAAGCCAG	GGCCTCAGAATCCATGCAAG	qRT‐PCR
METTL14	GACGGGGACTTCATTCATGC	CCAGCCTGGTCGAATTGTAC	qRT‐PCR
FTO	AGACACCTGGTTTGGCGATA	CCAAGGTTCCTGTTGAGCAC	qRT‐PCR
ALKBH5	ACCCCATCCACATCTTCGAG	CTTGATGTCCTGAGGCCGTA	qRT‐PCR
GAPDH	TCTCCTCTGACTTCAACAGCGACA	CCCTGTTGCTGTAGCCAAATTCGT	qRT‐PCR
GAPDH	TGAAGGTCGGAGTCAACGGATTTGGT	CATGTGGGCCATGAGGTCCACCAC	RT‐PCR
MyD88S	GACCCAGCATTGGGC	TCAGGGCAGGGACAAGGCCTTGGCAAG	RT‐PCR
MyD88L/S	GAGACACAAGCGGACCCC	CTGTTCCAGTTGCCGGATCA	RT‐PCR

### Western blot analysis

HDPCs were harvested using radioimmunoprecipitation assay (RIPA) lysis buffer (Beyotime, Haimen, China) and incubated on ice for 30 min. The protein concentration was measured using a bicinchoninic acid (BCA) protein assay (Beyotime). Forty micrograms of protein was separated by 10% sodium dodecyl sulphate–polyacrylamide gel electrophoresis (SDS‐PAGE) and electrophoretically transferred onto a polyvinylidene fluoride (PVDF) membrane (Millipore, Billerica, MA, USA). The membrane was blocked in TBST containing 5% non‐fat milk at room temperature for 1 hr and then incubated with the primary antibodies METTL3 (1:1000; Proteintech, Chicago, IL, USA), p65, p‐p65, IKKα, IKKβ, p‐IKKα/β, IκBα, p‐IκBα, ERK, p‐ERK, JNK, p‐JNK, p38, p‐p38, GAPDH and vinculin (1:1000; Cell Signaling Technologies, Danvers, MA, USA) overnight at 4°C. After the membrane was washed, it was incubated with secondary antibodies (Abcam, Cambridge, UK) for 1 hr at room temperature. Antibody binding was developed with an enhanced chemiluminescence system (Millipore) and scanned using an ImageQuant LAS 4000 mini system (GE Healthcare Life Sciences, Illinois, NJ, USA). Band densities were quantified and normalized to GAPDH or vinculin using ImageJ v1.47 software (National Institutes of Health, Bethesda, MD, USA).

### Cytokine assay and enzyme‐linked immunosorbent assay (ELISA)

The detection of cytokines and chemokines in cell culture supernatants obtained from different groups was performed with RayBio Human Cytokine Antibody Array 3 (RayBiotech, Norcross, GA, USA) in accordance with the manufacturer's instructions.

The concentrations of IL‐6, IL‐8, GRO, Gro‐α and RANTES in the cell culture supernatants were analysed using ELISA kits (RayBiotech) according to the manufacturer's instructions. Optical density (OD value) was measured at 450 nm using a microplate reader (Sunrise, Tecan, Switzerland).

### RNA sequencing

For the gene expression analysis, approximately 20 ng Poly (A) RNA was purified from total RNA and then converted to double‐stranded cDNA, and cDNA samples were sequenced with a HiSeq 2000 system (Illumina Inc., San Diego, California, USA). The sequencing reads were mapped to the human genome (hg19) using TopHat (v2.0.4). The expression levels of transcripts were quantified as reads per kilobase per million reads (RPKM). Differential genes were called at twofold changes using RPKM. Gene Ontology (GO) and Kyoto Encyclopedia of Genes and Genomes (KEGG) analyses were performed with the Database for Annotation, Visualization and Integrated Discovery (DAVID: http://david.abcc.ncifcrf.gov/).

For the splicing analysis, TopHat was used to identify the alignment of the reads onto the reference genome (hg19) and splice site, with mapping allowing up to two mismatches. The aligned reads were assembled into transcripts using Cufflinks software (v2.2.1). Cufflinks was used to compute normalized values termed fragments per kilobase of exon per million fragments mapped (FPKM), which reflect the mRNA expression levels. Statistical analysis of differentially expressed genes and transcript splice variants was performed with Cuffdiff (v2.2.1), which is integrated into Cufflinks. In the differential splicing analysis, Cuffdiff was used to calculate the changes in the relative abundance of splice variants produced from a single primary transcript.

### Statistical analysis

Each experiment was performed at least in triplicate. The data were expressed as mean ± S.E.M. Comparisons were analysed by Student's *t* test for two groups and by anova for multiple groups using SPSS v20.0 (SPSS, Inc., Chicago, IL, USA). The significance level was set at *P *<* *0.05.

## Results

### The total m6A content and METTL3 expression level are up‐regulated in LPS‐treated HDPCs

**Figure 1:**
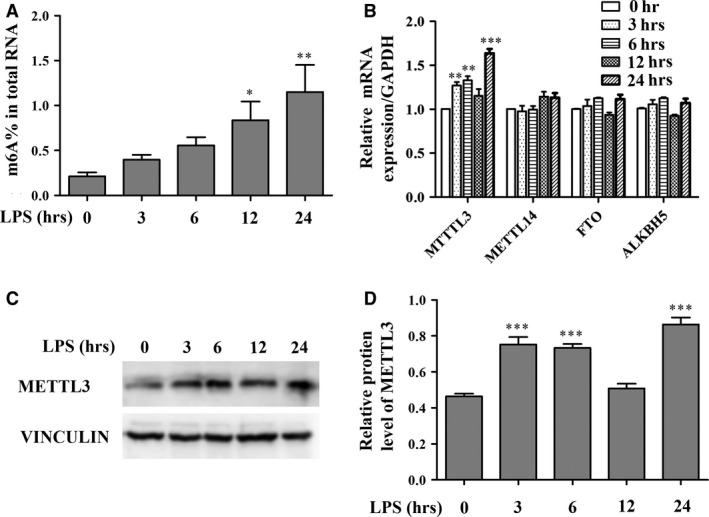
The total m6A content and METTL3 expression levels were increased in LPS‐treated HDPCs. HDPCs were treated with 1 μg/ml LPS for 3, 6, 12 and 24 hrs, and cells without LPS stimulation were used as controls. (**A**) The total m6A content was detected by quantifying m6A RNA methylation. (**B**) qPCR was performed to determine the mRNA expression levels of METTL3, METTL14, FTO and ALKBH5 in LPS‐treated HDPCs. GAPDH was used as an internal control. (**C, D**) METTL3 protein expression was assessed by Western blotting. Vinculin was used as an internal control. The data are expressed as the mean ± S.E.M. (*n* = 3). Statistically significant difference relative to the control (time 0), *P <* *0.05; **P <* *0.01; ***P <* *0.001.

To investigate the effect of LPS stimulation on the levels of m6A RNA modification in the total cellular RNA pools in HDPCs, quantification of m6A RNA methylation was performed. The result indicated that the m6A content was very low in HDPCs without LPS stimulation but increased in a time‐dependent manner after stimulation (Fig. 1A). Next, qPCR was performed to analyse the mRNA levels of m6A methyltransferase (METTL3, METTL14) and demethylases (FTO, ALKBH5). The METTL3 mRNA expression level increased after 3 hrs, reached a peak at 6 hrs, subsequently decreased slightly after 12 hrs and then rose again at 24 hrs after LPS treatment. METTL14, FTO and ALKBH5 mRNA expression levels did not significantly differ before and after LPS treatment (Fig. 1B). Similar up‐regulation of METTL3 protein expression was also observed using Western blotting (Fig. 1C and D). These findings suggest that the total m6A content and the METTL3 expression level are up‐regulated in LPS‐treated HDPCs.

### METTL3 inhibition down‐regulates the LPS‐induced expression of inflammatory cytokines in HDPCs

**Figure 2:**
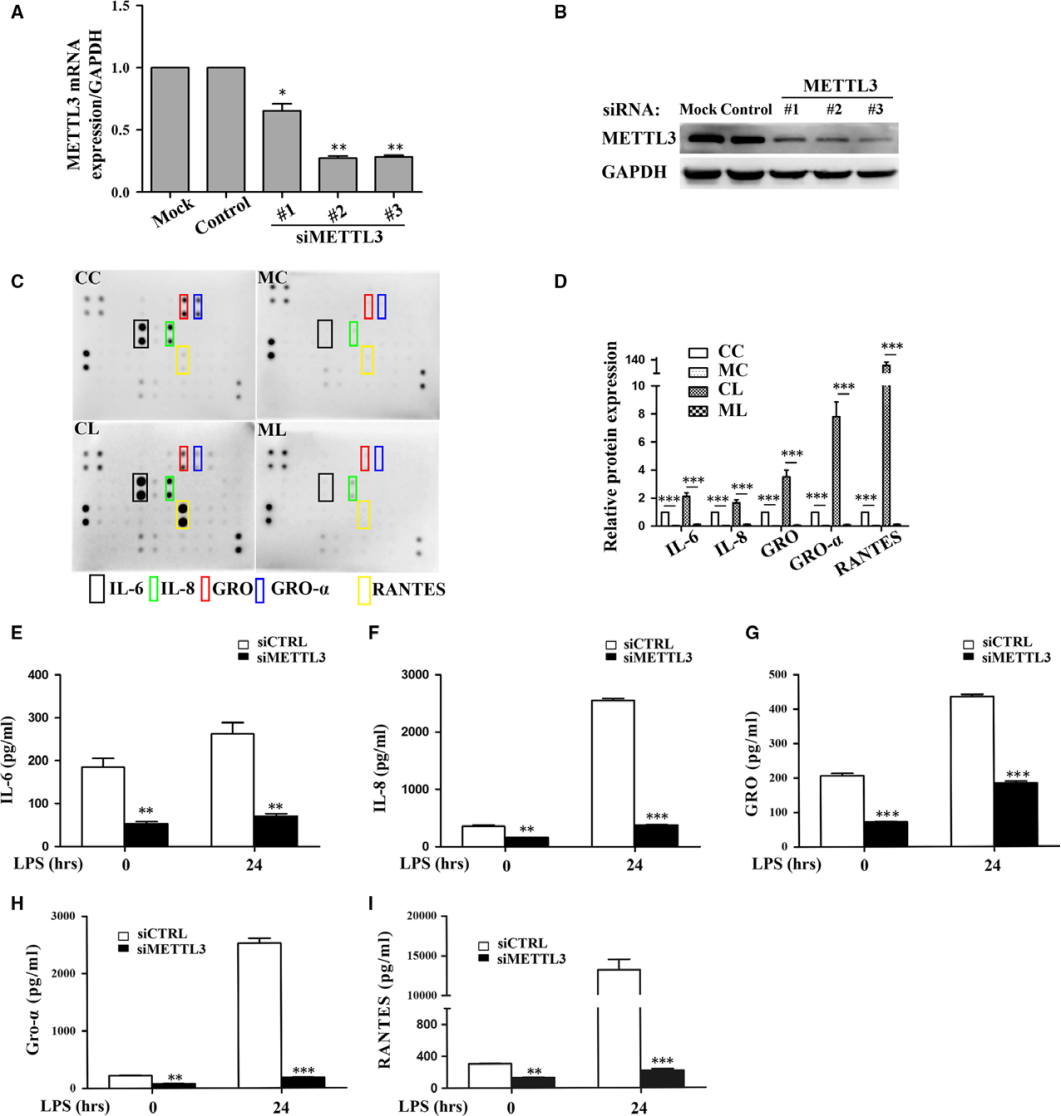
METTL3 depletion down‐regulated cytokine expression in LPS‐treated HDPCs. METTL3 knockdown in transfected HDPCs was confirmed by qPCR (**A**) and immunoblotting (**B**). Mock, cells transfected with only transfection reagent; Control, cells transfected with negative control siRNA; siMETTL3, cells transfected with METTL3 siRNA. (**C, D**) HDPCs were transfected with siRNAs (METTL3 or the negative control) and then treated with or without 1 μg/ml LPS for 24 hrs. The supernatant was collected and examined using a cytokine array. CC, cells transfected with negative control siRNA; CL, cells transfected with negative control siRNA and treated with LPS; MC, cells transfected with METTL3 siRNA; ML, cells transfected with METTL3 siRNA and treated with LPS. (**E–I**) The supernatants were collected, and the expression of IL‐6, IL‐8, GRO, Gro‐α and RANTES proteins was assessed by ELISA. siCTRL, cells transfected with negative control siRNA; siMETTL3, cells transfected with METTL3 siRNA. The data are presented as the mean ± S.E.M. (*n* = 3). Statistically significant difference relative to the siCTRL group (*P <* *0.05; **P <* *0.01; ***P <* *0.001).

To explore the role of METTL3 in the LPS‐induced inflammatory response of HDPCs, cells were transfected with METTL3 siRNA. METTL3 mRNA and protein expression levels were significantly decreased after METTL3 knockdown, whereas transfection with a control scramble siRNA had no effect (Fig. 2A and B). The cytokine antibody arrays were then used to examine the levels of 42 cytokines related to immunity and inflammation. As shown in Figure 2C and D, METTL3 knockdown decreased IL‐6, IL‐8, GRO, Gro‐α and RANTES expression levels compared with those in the siCTRL group before and after LPS stimulation. Their protein expression levels were verified by ELISA, and the results were consistent with those detected by the cytokine arrays (Fig. 2E–I).

### METTL3 inhibition down‐regulates LPS‐induced NF‐κB and MAPK signalling pathway activation in HDPCs

**Figure 3:**
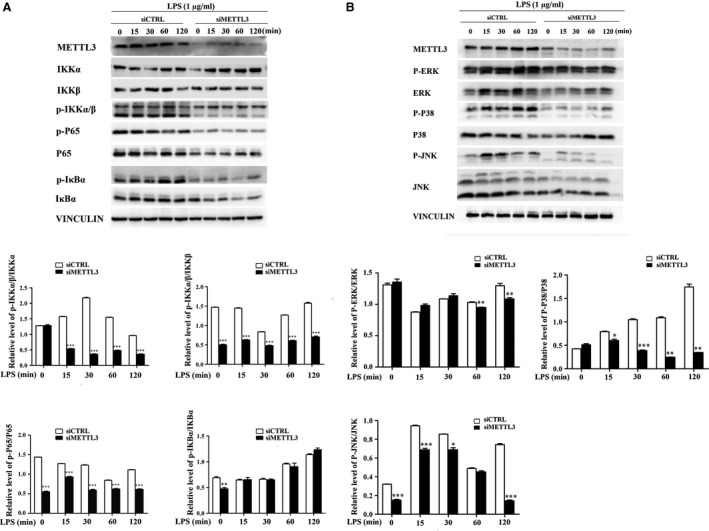
METTL3 inhibition down‐regulated LPS‐induced NF‐κB and MAPK signalling pathway activation in HDPCs. HDPCs were transfected with siRNAs (METTL3 or the negative control) and then treated with 1 μg/ml LPS for 0, 15, 30, 60 and 120 min. (**A**) The phosphorylation of IKKα, IKKβ, IκBα and p65 in the NF‐κB signalling pathway was examined by Western blotting. The relative quantitative analysis of the phosphorylation of IKKα, IKKβ, IκBα and p65 compared to that of the siCTRL group. (**B**) The phosphorylation of JNK, ERK and p38 in the MAPK signalling pathway was examined by Western blotting. The relative quantitative analysis of the phosphorylation of JNK, ERK and p38 compared to that of the siCTRL group. siCTRL, cells transfected with negative control siRNA; siMETTL3, cells transfected with METTL3 siRNA. The data are presented as the mean ± S.E.M. (*n* = 3). *P <* *0.05; **P <* *0.01; ***P <* *0.001.

To identify whether METTL3 depletion affects LPS‐induced NF‐κB and MAPK signalling pathway activation, Western blotting was performed to evaluate the phosphorylation levels of IKKα, IKKβ, p65, IκBα, p38, ERK and JNK. The results indicated that METTL3 knockdown remarkably reduced the levels of p‐IKKα/β, p‐p65 and p‐IκBα compared with those in the control group (Fig. 3A). The phosphorylation levels of p38, ERK and JNK were also down‐regulated in the METTL3‐knockdown cells (Fig. 3B). These data indicate that METTL3 can regulate NF‐κB and MAPK signalling pathway activation in HDPCs with and without LPS stimulation.

### METTL3 regulates the LPS‐induced inflammatory response of HDPCs

**Figure 4:**
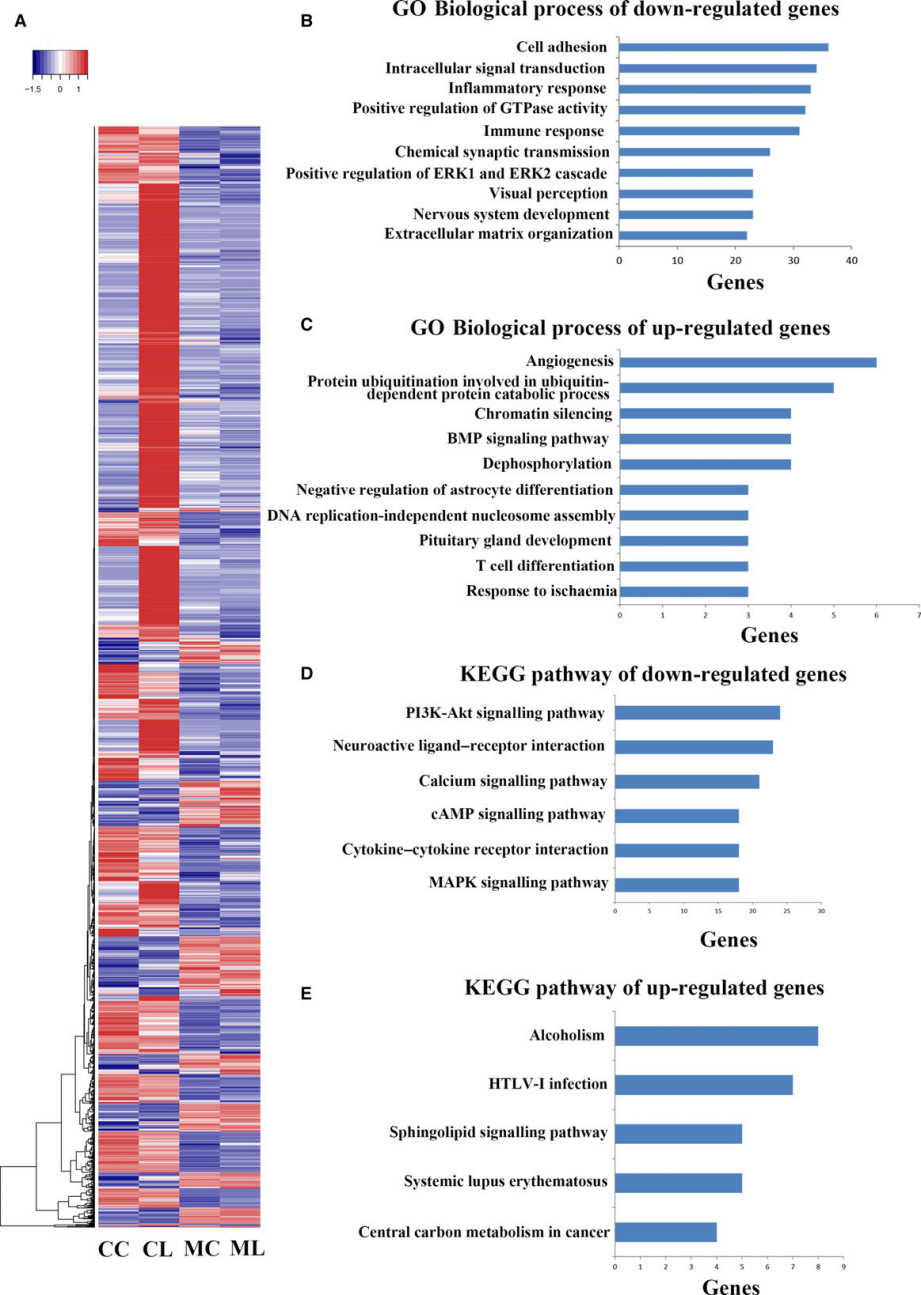
METTL3 inhibition down‐regulated the LPS‐induced inflammatory response of HDPCs. Total RNA obtained from the siCTRL and siMETTL3 cells with or without LPS treatment for 24 hrs was subjected to RNA sequencing. (**A**) The genes that were differentially expressed following LPS treatment or following METTL3 knockdown (MC *vs*. CC, or ML *vs*. CL) with a Log2 ratio >1.5 or <−1.5 are shown in the heatmap. CC, cells transfected with negative control siRNA; CL, cells transfected with negative control siRNA and treated with LPS; MC, cells transfected with METTL3 siRNA; ML, cells transfected with METTL3 siRNA and treated with LPS. Gene Ontology (**B**) and KEGG pathway (**D**) analyses of genes that were differentially expressed with a Log2 ratio <−1.5 ordered according to gene enrichment. Gene Ontology (**C**) and KEGG pathway (**E**) analyses of genes that were differentially expressed with a Log2 ratio >1.5 ordered according to gene enrichment.

To further determine the effect of METTL3 on dental pulp inflammation, siMETTL3 and siCTRL cells treated by LPS stimulation were subject to RNA sequencing. The genes that were differentially expressed following treatment with LPS or following METTL3 knockdown (siCTRL *vs*. siMETTL3 or siCTRL + LPS *vs*. siMETTL3 + LPS) with the Log2 ratio >1.5 or <−1.5 are shown in the heatmap (Fig. 4A). Furthermore, GO enrichment and KEGG pathway analyses were performed to organize the biological processes and pathways that were differentially activated between siMETTL3 and siCTRL cells upon LPS treatment. In the biological process analysis of genes down‐regulated by METTL3 depletion, the significant GO term was signal transduction with 57 transcripts enriched, followed by GO terms related to cell adhesion, intracellular signal transduction, inflammatory response, positive regulation of GTPase activity and immune response (Fig. 4B). In the biological process analysis of genes up‐regulated by METTL3 depletion, the significant GO terms were angiogenesis and protein ubiquitination involved in ubiquitin‐dependent protein catabolic process (Fig. 4C). KEGG pathway analysis of genes down‐regulated by METTL3 depletion revealed that the significant pathways were the PI3K‐Akt signalling pathway, neuroactive ligand–receptor interaction, calcium signalling pathway, cAMP signalling pathway, cytokine–cytokine receptor interaction and MAPK signalling pathway (Fig. 4D). KEGG pathway analysis of genes up‐regulated by METTL3 depletion showed that the significant pathways were alcoholism and human T‐lymphotropic virus type 1 (HTLV‐I) infection (Fig. 4E). These data imply that METTL3 knockdown affects the LPS‐induced inflammatory response in HDPCs.

### METTL3 regulates the alternative splicing of MyD88 in LPS‐stimulated HDPCs

**Figure 5:**
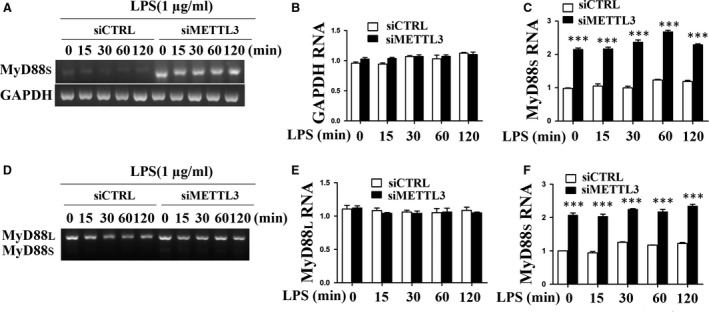
METTL3 inhibition facilitated MyD88S mRNA expression. HDPCs were transfected with siRNAs (METTL3 or the negative control) and then treated with 1 μg/ml LPS for 0, 15, 30, 60 and 120 min. (**A**) RT‐PCR was performed to amplify an mRNA product specific to MyD88S or mRNA product specific to GAPDH. Quantitation of the bands is depicted in (**B**) GAPDH and (**C**) MyD88S. (**D**) RT‐PCR was performed to amplify products from both MyD88L and MyD88S using primers that bracketed MyD88 exon 2. Quantitation of the bands is depicted in (**E**) MyD88L and (**F**) MyD88S. siCTRL, cells transfected with negative control siRNA; siMETTL3, cells transfected with METTL3 siRNA. The data are presented as the mean ± SEM (*n* = 3). Statistically significant difference relative to the siCTRL group (*P <* *0.05; **P <* *0.01; ***P <* *0.001).

Exon skipping in transcript isoforms is the most frequent event in alternative splicing 29. In our RNA sequencing data, exon skipping accounted for 39.13% of the total alternative splicing events in HDPCs. A previous study reported that the alternative splicing of MyD88 affects the inflammatory response 23. The splicing analysis herein revealed that MyD88 produced two isoforms, namely MyD88L and MyD88S, and that the expression of MyD88S, which lacks the second exon, was significantly increased by 1.3‐fold in the siMETTL3 group compared with that in the control group in LPS‐treated HDPCs (Table 2). To further confirm this result, RT‐PCR was performed to determine the mRNA levels of MyD88L and MyD88S. For these experiments, two sets of primers were used: One set was designed to specifically monitor MyD88S levels, and the other set was designed to monitor both MyD88L and MyD88S simultaneously. For the MyD88S‐specific primers, we used a reverse primer that spanned exons 3 to 1 and a forward primer that annealed to exon 1. For the primers that amplify both MyD88L and MyD88S, we used primers that annealed to exons 1 and 3 23. Using two sets of primers, we found that METTL3 inhibition did not significantly alter MyD88L mRNA levels but did significantly increase MyD88S mRNA levels (Fig. 5), consistent with the RNA sequencing results.

**Table 2:** Different expression analyses of transcript splice variants

Gene ID	Transcript	FRKM CL	FRKM ML	Fold Change	Official full name	
MYD88L	NM_002468	15.5842	13.008	−0.83	Myeloid differentiation primary response 88	Splice variant 2
MYD88S	NM_001172568	0.674897	0.887296	1.31	Myeloid differentiation primary response 88	Splice variant 3

CL, cells transfected with negative control siRNA and treated with LPS; ML, cells transfected with METTL3 siRNA and treated with LPS.

## Discussion

N6‐methyladenosine is a prevalent epitranscriptomic modification in mRNAs of most eukaryotes. This modification is widely conserved across plants and vertebrates and is also found in viruses and single‐cell organisms, such as archaea, bacteria and yeast 30, 31, 32, 33, 34, 35, 36. Although its existence was first reported 40 years ago 2, the biological function and significance of m6A modification have only recently entered the research spotlight. The abundance of m6A has been shown to be approximately 0.1–0.4% of the total adenosine residues in cellular mRNA, and the average content of m6A has been estimated to be three to five residues per mammalian mRNA 10. The m6A methylation is formed during nascent pre‐mRNA processing by a methyltransferase complex consisting of METTL3, METTL14 and WTAP and removed by FTO and ALKBH5 37, 38, 39, 40, 41. This modification plays important roles in neuronal disorders, immune response, obesity and cancer 42. The m6A modification is implicated in neurodegeneration and its associated disorders, for example Parkinson's disease 43. IL‐6 and IL‐8 have been demonstrated to be induced by dopamine, which is strongly influenced by m6A modification events in keratinocytes 44. An *in vivo* study using mouse models demonstrated that knocking down FTO results in reduced body weight and fat mass 45. METTL3 or METTL14 knockdown dramatically promotes human glioblastoma stem cell growth, self‐renewal and tumorigenesis 46. Few studies have offered insights into the role of m6A‐dependent RNA methylation in the development of oral inflammatory diseases thus far.

Dental pulp inflammation is bacterially driven inflammation of the dental pulp that occurs mainly due to the invasion of bacterial components and by‐products. Previous studies have shown that *Porphyromonas gingivalis* (*Pg*) is closely related to dental pulp inflammation 13, 14. *Pg* LPS is regarded as an inflammatory stimulator and is therefore used to establish models of dental pulp inflammation and periodontal disease 14, 47, 48. To explore the role of m6A‐dependent RNA methylation in dental pulp inflammation, we used *Pg* LPS in the present study to establish a model of dental pulp inflammation, and we detected the m6A content and expression levels of m6A methyltransferase and demethylases, including METTL3, METTL14, FTO and ALKBH5. The results showed that the m6A level was very low in HDPCs before LPS treatment but increased in a time‐dependent manner after LPS stimulation. METTL3 mRNA and protein levels increased after LPS stimulation, whereas METTL14, FTO and ALKBH5 expression levels did not significantly differ with or without LPS stimulation. These findings indicated that METTL3‐dependent m6A modification might be involved in the LPS‐induced inflammatory response of HDPCs.

METTL3 (also known as MTA70) was originally identified as a methyltransferase responsible for m6A modification in mRNA 49. Depletion of the *Arabidopsis thaliana* METTL3 homologue (MTA) during later growth stages gives rise to plants with altered growth patterns and reduced apical dominance 50, and inhibition of the *Drosophila* METTL3 homologue (Dm ime4) has been shown to inhibit oogenesis 51. In zebrafish, METTL3 knockdown leads to smaller heads, eyes and brain ventricles, as well as curved notochords 38. Mettl3 knockdown and Mettl14 knockdown in murine embryonic stem cells (ESCs) result in reduced m6A abundance and defective cell regeneration 52. METTL3 inhibition leads to apoptosis of human HeLa cells and a concomitant decrease in cellular m6A levels 9. To identify whether METTL3 affects the LPS‐induced inflammatory responses of HDPCs, we inhibited METTL3 in HDPCs and found that METTL3 knockdown reduced the accumulation of inflammatory cytokines such as IL‐6 and IL‐8. The phosphorylation levels of IKKα, IKKβ, p65, IκBα, p38, ERK and JNK in the NF‐κB and MAPK signalling pathways were also decreased in the METTL3‐knockdown cells. Surprisingly, the expression of IκBα was down‐regulated when the NF‐κB signalling pathway was deactivated, which was not consistent with previous studies 53, 54. m6A modification is reported to affect pre‐mRNA splicing, mRNA stability and protein translation 10, 55. We suspect that METTL3 might affect IκBα mRNA stability and consequent degradation, or its translation, which should be further studied. The RNA sequencing results suggested that many genes were regulated by METTL3 depletion. The GO term analysis indicated that many of the down‐regulated genes were associated with the inflammatory response and immune response. KEGG pathway analysis revealed that the signalling pathways associated with the down‐regulated genes were mostly involved in regulating inflammation. The above data suggested that METTL3 can regulate the LPS‐induced inflammatory responses in HDPCs.

Alternative splicing can generate a range of mRNA transcripts and protein isoforms from a limited number of genes, which enables the synthesis of diverse isoforms with different or even opposite functions 56. More than 90% of human genes undergo alternative splicing, which is especially prevalent in the immune system 57. Alternative splicing can regulate intracellular signalling and intercellular communication through the expression of diverse isoforms of cytokines, cytokine receptors, kinases, phosphatases and adaptor proteins 58. Myd88s, a splice variant of MyD88 lacking the second exon, can function as a negative regulator of TLR signalling and limit the extent of innate immune activation 23. METTL3 localizes predominantly in nuclear speckles, which are sites of mRNA splicing and storage 38. METTL3 depletion in mouse ESCs generally facilitates exon skipping and intron retention 4, and METTL3 knockdown has an effect on the alternative splicing patterns of genes in the p53 signalling pathway, resulting in cell apoptosis 5. These studies imply that METTL3 plays an important role in regulating alternative splicing. In the present study, the splicing analysis revealed that the expression of the alternative spice variant of MyD88 (MyD88S) was up‐regulated 1.3‐fold in METTL3‐depleted HDPCs compared with that in the control group after LPS stimulation. This result inspired us to hypothesize that METTL3 might regulate the alternative splicing of MyD88 in LPS‐induced HDPCs. To address this hypothesis, we determined the expression level of MyD88S using RT‐PCR. The result indicated that METTL3 knockdown significantly promoted MyD88S mRNA expression, consistent with the RNA sequencing result. Previous studies have shown that MyD88S cannot recruit IRAK‐4 and fails to activate the NF‐κB signalling pathway 21, 22, 59. Our findings suggested that METTL3 might affect the LPS‐induced inflammatory response of HDPCs by regulating the alternative splicing of MyD88. METTL3 also affected cytokine expression and the NF‐κB and MAPK signalling pathways without LPS stimulation (time 0). MyD88s showed basal expression, but the expression was very low in the control group and increased in the METTL3‐depleted cells without LPS treatment. Therefore, we speculated that METTL3 might affect the expression of cytokines and the NF‐κB and MAPK signalling pathways by regulating the alternative splicing of MyD88 even without LPS stimulation.

In summary, we reported that m6A and METTL3 expression levels were up‐regulated in LPS‐stimulated HDPCs and that METTL3 depletion decreased the accumulation of inflammatory cytokines and suppressed the activation of the NF‐κB and MAPK signalling pathways. Moreover, our results demonstrated that METTL3 modulated the alternative splicing of MyD88, suggesting that METTL3 might affect the LPS‐induced inflammatory response by regulating the alternative splicing of MyD88 in HDPCs. These findings might contribute to novel progress in the role of the epitranscriptome in the inflammatory response and provide a promising molecular mechanism for new therapeutic strategies for treating dental pulp inflammation.

## Conflict of interest

The authors confirm that there is no conflict of interests.
